# Changes in the Transcriptome Caused by Mutations in the Ribosomal Protein uS10 Associated with a Predisposition to Colorectal Cancer

**DOI:** 10.3390/ijms23116174

**Published:** 2022-05-31

**Authors:** Yueming Tian, Elena S. Babaylova, Alexander V. Gopanenko, Alexey E. Tupikin, Marsel R. Kabilov, Alexey A. Malygin, Galina G. Karpova

**Affiliations:** 1 Institute of Chemical Biology and Fundamental Medicine, Siberian Branch of the Russian Academy of Sciences, Novosibirsk 630090, Russia; moon20160324@163.com (Y.T.); lalena2005@ngs.ru (E.S.B.); alexandr.gopanenko@yandex.ru (A.V.G.); alenare@niboch.nsc.ru (A.E.T.); kabilov@niboch.nsc.ru (M.R.K.); malygin@niboch.nsc.ru (A.A.M.); 2 Department of Molecular Biology, Novosibirsk State University, Novosibirsk 630090, Russia

**Keywords:** HEK293T cells, mutations in human ribosomal protein uS10, predisposition to colorectal cancer, next generation sequencing, differently expressed genes, uS10 mutation-dependent genes

## Abstract

A number of mutations in the *RPS20* gene encoding the ribosomal protein uS10 have been found to be associated with a predisposition to hereditary non-polyposis colorectal carcinoma (CRC). We transfected HEK293T cells with constructs carrying the uS10 minigene with mutations identical to those mentioned above and examined the effects of the produced proteins on the cellular transcriptome. We showed that uS10 with mutations p.V50SfsX23 or p.L61EfsX11 cannot be incorporated into 40S ribosomal subunits, while the protein with the missense mutation p.V54L functionally replaces the respective endogenous protein in the 40S subunit assembly and the translation process. The comparison of RNA-seq data obtained from cells producing aberrant forms of uS10 with data for those producing the wild-type protein revealed overlapping sets of upregulated and downregulated differently expressed genes (DEGs) related to several pathways. Among the limited number of upregulated DEGs, there were genes directly associated with the progression of CRC, e.g., *PPM1D* and *PIGN*. Our findings indicate that the accumulation of the mutant forms of uS10 triggers a cascade of cellular events, similar to that which is triggered when the cell responds to a large number of erroneous proteins, suggesting that this may increase the risk of cancer.

## 1. Introduction

Ribosomes are cellular nanomachineries required for the translation of genetic information coming to them as messenger RNAs (mRNAs) into the polypeptide chains of proteins and are composed of large and small subunits that both consist of RNA molecules and several dozen proteins. The mammalian ribosome contains 80 proteins that are assembled together during the biogenesis of 60S and 40S ribosomal subunits, a complex and energy-intensive process that starts in the nucleolus, continues in the nucleoplasm and ends in the cytoplasm [1,2]. In mature subunits, ribosomal proteins are involved not only in maintaining translationally competent subunit structures, but also in the formation of ribosomal functional centers, providing both conservative and eukaryote/archaea-specific interactions of subunits and/or ribosomes with their ligands, such as mRNA, tRNA and translation factors [3,4,5,6,7,8]. Defects in ribosome biogenesis and protein synthesis lead to various abnormalities called ribosomopathies [9], which have recently come to be considered as a risk factor for cancer initiation [10]. Furthermore, ribosomal proteins are known to perform so-called extra-ribosomal functions in a wide range of processes not directly related to their participation in translation as components of the ribosome [7,11,12,13]. The disruption of these functions of ribosomal proteins may also result in cellular abnormalities, which are a risk factor for cancer. Obviously, all these disorders can be caused by mutations in the genes of ribosomal proteins, whose occurrence in a number of such genes has been discovered in various types of cancer by recent studies using whole-exome or whole-genome sequencing techniques, as reviewed in [10]. Among these genes is *RPS20*, which encodes the ribosomal protein uS10 (previously named as S20), mutations of which are associated with colorectal cancer [14,15,16]. This protein is located on the solvent-exposed surface of the head of the 40S subunit adjacent to the mRNA entry site and interacts directly with the ribosomal protein uS3 [17,18], which is also involved in the interaction with the translation initiation factor eIF3, as shown for yeast ribosomes [19]. Both proteins bind to the pre-40S subunit at the final stages of its maturation, and uS10 immediately interacts with assembly factors orchestrating maturation events across the pre-40S subunit, which makes it an indispensable participant in this process [20].

Among the functional assignments of the ribosome-associated protein uS10 is its involvement in the processes of ribosome-mediated quality control (RQC) through ubiquitination [21]. The protein also becomes a target for ubiquitination in ribosomes stuck on poly(A)-sequences encoding polylysine, which triggers the degradation of the stalled ribosomes by the RQC system [22,23], whose key player is the E3 ubiquitin protein ligase (Hel2 in yeasts [22] and ZNF598 in mammals [23]). Similarly, when the translation of mRNA is stopped due to the collision of ribosomes, their degradation is initiated by the no-go decay (NGD) mechanism, in which the uS10 ubiquitination also plays a central role [24].

Ribosome-free uS10 is able to engage in interactions with a number of other cellular partners, due to which it can participate in processes that control cell proliferation. In particular, the protein has been found to bind to the E3 ubiquitin ligase Mdm2 and its homologue MdmX and thus to be involved in the regulation of the Mdm2-p53-MdmX network that is crucial for ribosome biogenesis surveillance [25]. Furthermore, it has recently been discovered that uS10 is an important partner of the nucleolar GTPase GNL1, and that the interaction between these proteins promotes the progression of the G1/S phase during the cell cycle and cell proliferation, causing the hyperphosphorylation of the retinoblastoma protein [26].

Given the involvement of uS10 in the control of translation and cell proliferation, it is not surprising that mutations in its gene can lead to various abnormalities in mammals. For example, in mice, a point mutation in the *RPS20* gene, corresponding to the L32P amino acid substitution in uS10, causes epidermal melanocytosis, resulting in a dark skin phenotype [27]. It has been shown that this mutation is characterized by the accumulation of p53 in cells and the increased expression of the *Kitl* gene, whose high expression in keratinocytes leads to the darkening of the skin [27]. The first evidence of the association of mutations in the *RPS20* gene with human diseases has been reported in the study [14], from which it becomes clear that they are related to a predisposition to hereditary non-polyposis colorectal carcinoma (CRC). A little later, a linkage of mutations in uS10 with CRC susceptibility has been confirmed for such disruptive mutations as p.V50SfsX23 and p.L61EfsX11, and for the missense one p.V54L, by the evidence reviewed in [15]. More recently, another uS10 missense mutation, p.E33V, has been shown to be associated with an inherited predisposition to several types of cancers, including CRC [16]. Finally, two mutant variants of the *RPS20* gene have recently been described that correspond to substitutions I84N and I84S in the protein in patients with the genetic disorder, Diamond-Blackfan anemia (DBA), a form of ribosomopathy characterized by red cell aplasia and macrocytic anemia [28] and usually caused by mutations in genes of a number of ribosomal proteins [29]. Although the list of diseases whose initiation and progression could be affected by mutations in the *RPS20* gene is expanding, it remains unknown what changes in the transcriptome of mammalian cells usually occur when this gene is mutated.

Believing that, in general, the above changes should be basically similar for different mammalian cell types, we applied RNA-seq analysis to HEK293T cells ectopically producing the FLAG-tagged ribosomal protein uS10 carrying the p.V50SfsX23, p.L61EfsX11, or p.V54L mutations to determine their effects on gene expression at the transcriptional level. Analyzing the obtained data, we tried to find among the revealed effects those which may contribute to the malignant transformation of cells. We showed that the appearance of the above uS10 mutants in cells leads to a change in gene expression, manifested in the increased activity of certain sets of genes closely associated with carcinogenesis through the p53 pathway and with some other processes, including those related to the induction of cellular senescence. Our findings may explain the previously reported link between the mutations in the *RPS20* gene, corresponding to those in the ribosomal protein uS10 examined in the current study, and a predisposition to CRC. 

## 2. Results

### 2.1. The Ectopic Production of Mutant Forms of the Ribosomal Protein uS10 in HEK293T Cells

**Figure 1:**
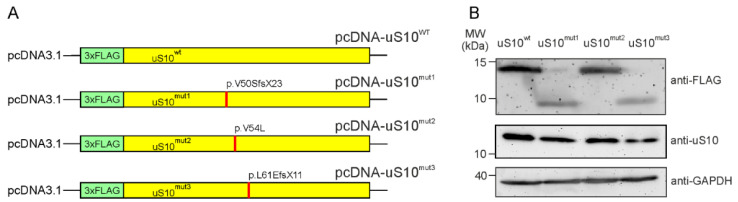
Plasmid constructs encoding human ribosomal protein uS10 and its mutant forms. (**A**) Maps of constructs encoding 3×FLAG-tagged target proteins, the wild type uS10 (uS10^wt^) and its forms with mutations identical to those related to a predisposition to CRC (uS10^mut1^, uS10^mut2^ and uS10^mut3^). The positions of the mutations are indicated with red lines and marked. (**B**) Western blot analysis of the production of exogenous proteins, endogenous uS10 and GAPDH as a reference in HEK293T cells transfected with the above plasmid constructs using the anti-FLAG M2, anti-uS10 and anti-GAPDH antibodies. Anti-uS10 antibodies bind to the endogenous protein but not to exogenous 3×FLAG-tagged uS10^wt^ and its mutant forms.

To obtain HEK293T cells producing exogenous human uS10, we cloned the insert containing the coding sequence (CDS) of the *RPS20* gene into the pcDNA3.1 mammalian expression vector under the CMV promoter. Since the N-terminus of uS10 is unstructured and extends away from the ribosome body [17,18], we assigned the N-terminal 3×FLAG epitope tag as a specific marker of the exogenous uS10. The resulting plasmid encoding 3×FLAG-tagged wild type (wt) uS10, pcDNA-uS10^wt^ was used for the transient transfection of HEK293T cells. To prepare plasmids encoding 3×FLAG-tagged mutant forms of uS10 (Figure 1A), the above was subjected to site-directed mutagenesis to introduce changes into the CDS, identical to the ones found in the *RPS20* gene in patients with a predisposition to hereditary CRC [15]. In particular, an insertion of the A residue into the Val50 codon converted it to the Ser one and caused a frameshift in the CDS downstream from the replacement site, resulting in the pcDNA-uS10^mut1^ plasmid encoding the truncated form of uS10, p.V50SfsX23 (uS10^mut1^). The replacement of the G residue with the C one in the Val54 codon changed it to the Leu codon, yielding the plasmid pcDNA-uS10^mut2^ encoding the full-length uS10 with the missense mutation, p.V54L (uS10^mut2^). Finally, a double deletion of TT in the Leu61 codon transformed it into Glu, leading to a shift in the reading frame, similar to that which occurred when the A was inserted into the Val50 codon, giving the plasmid pcDNA-uS10^mut3^ and encoding the truncated uS10 mutant, p.L61EfsX11 (uS10^mut3^). 

The production of target proteins by HEK293T cells transiently transfected with the resulting plasmid constructs was examined by Western blotting using anti-FLAG antibodies applied to total protein samples from whole cell lysates transferred onto the nitrocellulose membrane after SDS PAGE. All the FLAG-tagged proteins were shown to be ectopically produced in HEK293T cells, although the yields of the truncated protein forms, uS10^mut1^ and uS10^mut3^, were approximately half those of the full-length ones, uS10^wt^ and uS10^mut2^ (Figure 1B).

**Figure 2:**
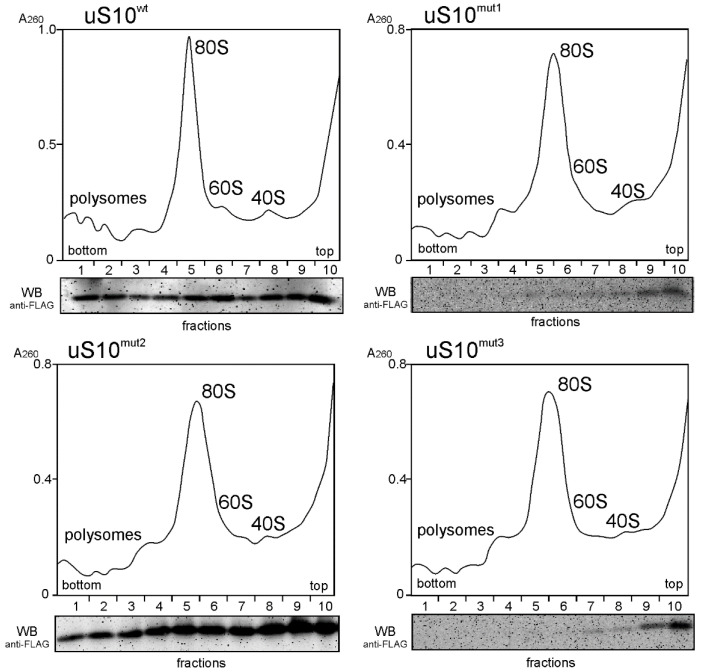
The polysome profiles from HEK293T cells producing 3×FLAG-tagged target proteins, uS10^wt^ or its mutated forms uS10^mut1^, uS10^mut2^ or uS10^mut3^, obtained by the centrifugation of their cytosolic extracts in a sucrose density gradient. Peaks corresponding to 40S and 60S subunits, 80S ribosomes and polysomes are marked. Below each panel, a Western blot analysis of the exogenous protein content in the gradient fractions using anti-FLAG antibodies is presented. The presence of signals from proteins uS10^mut1^ and uS10^mut3^ in fractions 5–9, weakening in the direction from the upper fractions to the lower ones, is due to some contamination of these fractions with the contents from the upper fractions, where these proteins are in large quantities.

To test the ability of the 3×FLAG-tagged exogenous proteins to participate in the assembly of 40S ribosomal subunits together with the functional competence of the subunits containing these proteins in translation, we examined the contents of uS10^wt^ and its mutant forms in fractions of polysome profiles from cytosolic extracts of the respective cells. Both uS10^wt^ and uS10^mut2^ with the p.V54L missense mutation were found to be present in the fractions of the 40S subunit, 80S ribosomes and polysomes, while the truncated protein forms, uS10^mut1^ and uS10^mut3^, were not included in any of these fractions (Figure 2). This proves that the missense mutation p.V54L does not prevent the exogenous protein uS10^mut2^ from replacing endogenous uS10 in the assembly of 40S subunits and that 80S ribosomes containing uS10^mut2^ are involved in translation. In contrast, mutations p.V50SfsX23 and p.L61EfsX11 are disruptive and lead to the loss of protein functionality; although both uS10^mut1^ and uS10^mut3^ are produced in HEK293T cells, their degradation should be more intense than that of uS10^wt^ and uS10^mut2^, which could explain the lower yields of the former two proteins compared to those of the latter two (Figure 1B).

### 2.2. A Comparative Analysis of RNA-Seq Data from Cells Producing 3×FLAG-Tagged uS10 and Its Mutant Forms

**Figure 3:**
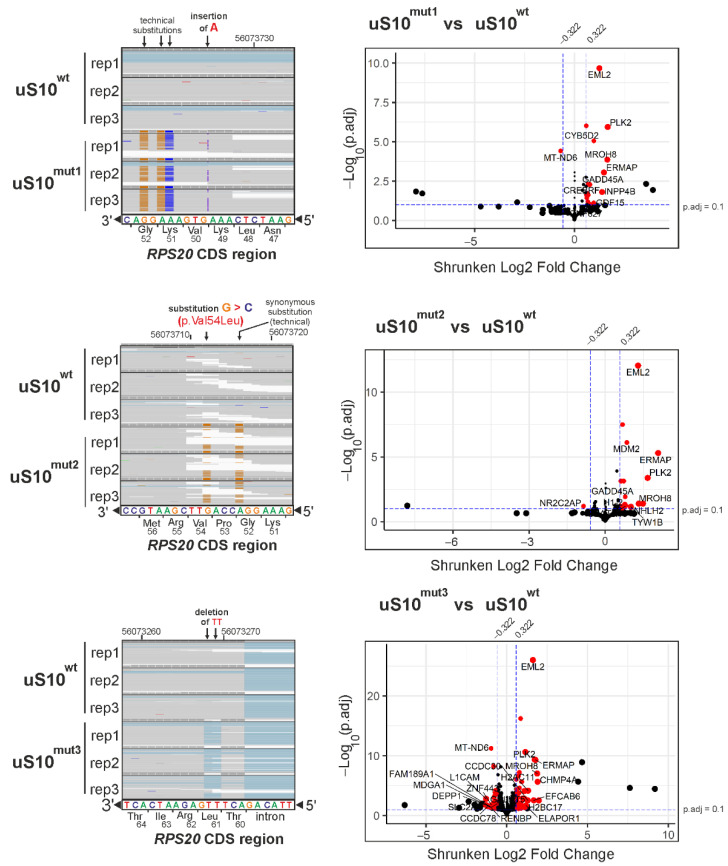
The next generation sequencing of total RNA samples from HEK293T cells producing 3×FLAG-tagged target proteins, uS10^wt^ or its mutated forms. Left panels. Representative fragments of the IGV genome browser view showing the sequencing read coverage of the range corresponding to the RPS20 gene region encoding the ribosomal protein uS10. The read mapping is displayed for three biological replicates of each sample. Positions of mutations are shown by arrows. Right panels. Volcano plots of the differentially expressed genes between cells producing uS10^mut1^, uS10^mut2^ or uS10^mut3^ and to uS10^wt^-producing ones. In the plots, upregulated and downregulated genes sorted according to the criteria specified in the Materials and Methods section (DEGs) are shown as red dots, and genes that do not meet these criteria are displayed as black ones. The dashed vertical and horizontal lines represent the cutoff values of shrunken log2 fold changes and a p adjusted (p.adj), respectively.

To reveal changes in the transcriptome of cells producing uS10 with mutations associated with a predisposition to CRC, we applied RNA-seq analysis to HEK293T cells transfected with the pcDNA-uS10^mut1^, pcDNA-uS10^mut2^ or pcDNA-uS10^mut3^ plasmids, and compared the gained results with those obtained on cells transfected with the non-mutant pcDNA-uS10^wt^ construct. To this end, total RNA samples were isolated from the cells after two days of treatment with the above constructs and subjected to polyA selection, followed by next generation sequencing (NGS). An analysis of the raw RNA-seq data using the FastQC tool (Figure S1) confirmed the high-quality of the RNA-seq data (Figure S2). After quality control of the raw data, the sequencing reads were filtered and mapped to the human genome. The metadata for all samples are provided in Table S1. The mapped reads predominantly fell within genes, rather than intergenic regions (Figure S3). Mapping revealed a more than 13-fold predominance of exogenous mRNA encoding uS10^wt^ or its forms with targeted mutations over mRNA encoding endogenous uS10 in transfected cells (Figure 3, left panels). The resulting gene sets were analyzed using the DESeq2 package (Table S2) and criteria allowing sorting genes with the most pronounced and statistically significant changes in expression levels in cells producing uS10^mut1^, uS10^mut2^ or uS10^mut3^, compared to uS10^wt^-producing ones (Figure 3, right panels). 

**Figure 4:**
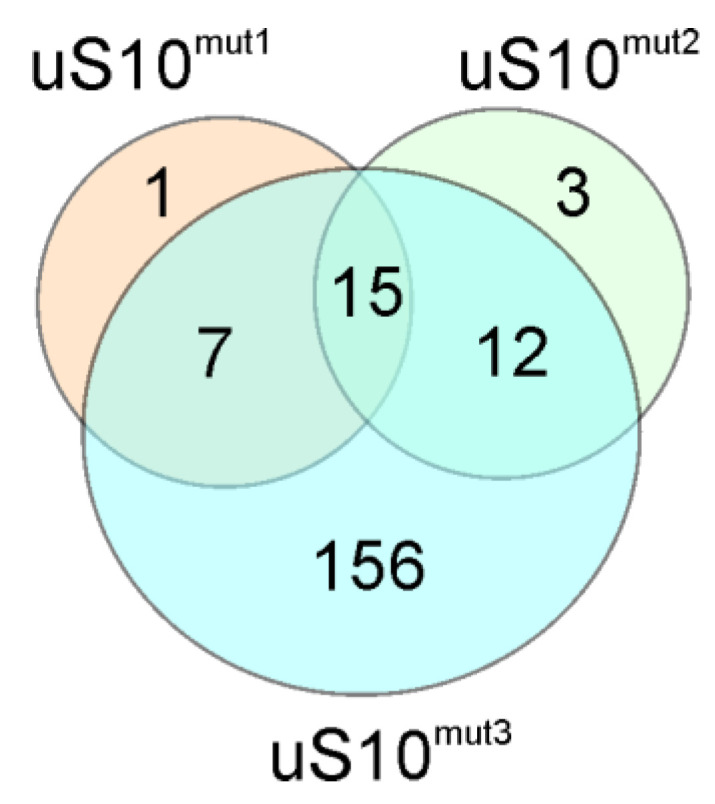
Venn diagram showing the overlap of upregulated DEGs for HEK293T cells producing mutant forms of uS10^wt^.

We found that in cells producing uS10^mut1^, the expression of 24 genes was altered, of which 23 were upregulated and 1 was downregulated, while in cells producing uS10^mut2^, the transcriptional efficiency of 35 genes changed, of which 30 were activated and 5 were suppressed. Finally, for cells producing uS10^mut3^, changes in expression levels of 390 genes were observed, with an increase for 190 genes and a decrease for 200 ones. The found sets of differently expressed genes (DEGs) are presented in Table S3. It is noteworthy that, despite the inability of the uS10^mut1^ and uS10^mut3^ proteins to integrate into the 40S subunit, unlike uS10^mut2^, which, in addition to being incorporated into the 40S subunit, can also participate as its component in the operation of the translation machine, the sets of upregulated DEGs found for cells producing these proteins overlap with the set for cells producing uS10^mut2^, forming a group of 15 shared DEGs (Figure 4). Among the DEGs that fell into this group, there were tumor-associated genes such as *GADD45A*, *PPM1D*, *PLK2* and *CREBRF* (Table S3). The signaling protein GADD45A (growth arrest and DNA damage-inducible protein alpha) encoded by the *GADD45A* gene plays many roles in mediating stress signaling and is thereby implicated in growth regulation, DNA demethylation, DNA damage, DNA repair, apoptosis and other processes (see for a review [30,31]). The transcription of *GADD45A* is regulated by p53, BRCA1 (breast cancer type 1 susceptibility protein) and c-myc in response to a variety of stimuli, making this gene an essential player in tumorigenesis (see for a review [32]). The *PPM1D* gene (also known as *WIP1*) encodes wild-type p53-induced phosphatase 1 protein, a negative regulator in the p53 signaling pathway, which has an important role in cellular stress responses, the regulation of the cell cycle and the metabolism of tumor cells, exerting its oncogenic function in various cancer forms by enhancing cell proliferation (see for a review [33,34] and references therein). For example, the high expression of *PPM1D* is associated with poor prognosis for the esophageal squamous cell carcinoma patients [35], whereas its silencing inhibits colon cancer cell proliferation [36]. The serine/threonine protein kinase PLK2 (polo-like kinase) encoded by the *PLK2* gene, which can also be induced by p53, acts as a putative tumor suppressor by interacting with signaling molecules involved in antitumor activity, while the dysregulation of *PLK2* in some types of cancer results in tumor-promoting effects (see [37] and refs. therein). As for the *CREBRF* gene encoding a negative regulator of the endoplasmic reticulum stress response, it can facilitate the proliferation of human gastric cancer cells [38] and plays a central role in the progression of malignant glioma [39]. 

Two p53 target genes (*MDM2* and *TP53INP1*) were found among upregulated DEGs in cells producing uS10^mut2^ and uS10^mut3^, and one such gene, *SPATA18*, was present in the set of the DEGs for cells producing uS10^mut2^ (Table S3). The *MDM2* gene encodes a proto-oncogene *MDM2*, which is an E3 ubiquitin ligase mediating the ubiquitination of a large group of proteins, including p53 and some other tumor suppressor proteins (see for a review [40]). The overexpression of *MDM2*, observed in many types of cancer, plays a key role in tumorigenesis (see, e.g., [41,42]). The *SPATA18* gene encodes a spermatogenesis associated protein 18, also known as MIEAP (mitochondria-eating protein), which is a central participant in mitochondrial quality control via mitophagy [43], a process that is enhanced in response to DNA damage [44]. The *TP53INP1* gene encodes the tumor protein p53-inducible nuclear protein 1, which is an antiproliferative and proapoptotic protein involved in cell responses to various stresses [45]. This gene plays a significant role in the progression of anaplastic carcinoma [46] and has been shown to be overexpressed in medullary thyroid carcinoma and prostate cancer [47,48]. 

In addition, the group of upregulated DEGs common to all uS10 mutants included genes such as *AEN* (apoptosis enhancing exonuclease), involved in p53-induced apoptosis following DNA damage, *BTG2* (BTG anti-proliferation factor 2), associated with the regulation of the cell cycle, and *HMMR* (hyaluronan-mediated motility receptor) and *ERMAP* (erythroblast membrane associated protein), encoding receptors related to cell motility and adhesion, respectively. In this group, there were also *EML2* (echinoderm microtubule associated protein like 2), linked to the inhibition of microtubule growth, *PIGN* (phosphatidylinositol glycan anchor biosynthesis class N), implicated in glycolipid biosynthesis and several others. 

As for downregulated DEGs, one gene (*MT-ND6*), found in cells producing uS10^mut1^ and the three genes (*CRTAP*, *NDRG1* and *NR2C2AP*), of the five ones discovered in cells producing uS10^mut2^ were also present in the set of the DEGs identified for uS10mut3-producing cells. The *MT-ND6* gene is a mitochondrial one that encodes NADH dehydrogenase subunit 6. The *CRTAP* gene encodes a cartilage-associated protein that is required for the hydroxylation of fibrillar collagen, and the *NR2C2AP* gene encodes a nuclear receptor 2C2-associated protein that can repress hormone receptor NR2C2. The product of the *NDRG1* gene is a stress-responsive protein that acts as a tumor suppressor. Finally, the set of uS10^mut3^-associated downregulated DEGs contain a large group of genes encoding collagens (*COL4A2*, *COL4A6*, *COL6A2*, *COL27A1*, *COL18A1* and *COL1A1*) and transporter proteins (*SLC37A4*, *SLC29A2*, *SLC19A1*, *SLC16A2*, *SLC25A29*, *SLC25A1* and *SLC29A4*). 

It should be noted that the transfection of cells with pcDNA-uS10^wt^ per se induced a cellular response accompanied by the upregulation or downregulation of about 400 genes compared to non-transfected cells (Table S4). Notably, this set contained several activated DEGs from sets of those found with cells producing uS10^mut1^, uS10^mut2^, or uS10^mut3^, including tumor-associated genes such as *GADD45A*, *PPM1D*, *MDM2*, and *CREBRF*. The expression levels of these DEGs in cells transfected with pcDNA-uS10^wt^ were approximately 1.4–1.8-times higher than in non-transfected cells (see the values in the shrunken log2 fold change (LFC) column in Table S4). However, their expression levels were increased by about the same factor in cells transfected with plasmids encoding the uS10 mutant forms compared to those in cells transfected with pcDNA-uS10^wt^ (see the values in the shrunken LFC column in Table S2). This meant that in cells producing the uS10^wt^ mutant forms, the above DEGs were activated much more strongly than in cells producing exogenous uS10^wt^. Notably, tumor-associated genes such as *PLK2* and *PIGN* were among the DEGs activated specifically in cells producing the uS10 mutants.

**Figure 5:**
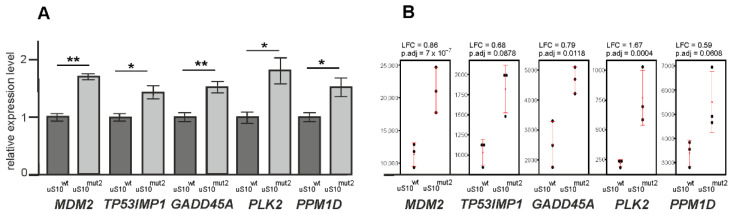
Validation of the results of the differential gene expression analysis with the RNA-seq data by RT-qPCR. (**A**) The RT-qPCR data obtained for selected upregulated DEGs with cells producing uS10^mut2^; columns show the relative levels of mRNAs (* *p* < 0.05, ** *p* < 0.01 (Mann–Whitney test)). (**B**) The results of the analysis of differential expression of the same genes obtained using RNA-seq data and shown as dotplots with mean ± SD values. Dots display the values of normalized read counts for each sample.

To validate the results of the differential gene expression analysis performed with the RNA-seq data, we carried out the same analysis with the RT-qPCR data obtained with HEK293T cells producing uS10^mut2^ for a group of selected genes, namely *GADD45A*, *PPM1D* and *PLK2*, upregulated in cells with uS10^mut1^, uS10^mut2^ or uS10^mut3^, and *MDM2* and *TP53INP1*, upregulated in cells with uS10^mut2^ or uS10^mut3^ (Figure 5). The values of changes in the expression levels of the above genes, estimated from RNA-seq and RT-qPCR data, were very similar.

### 2.3. Processes Associated with Genes Whose Expression Changes When Cells Produce Mutant Forms of uS10wt

**Figure 6:**
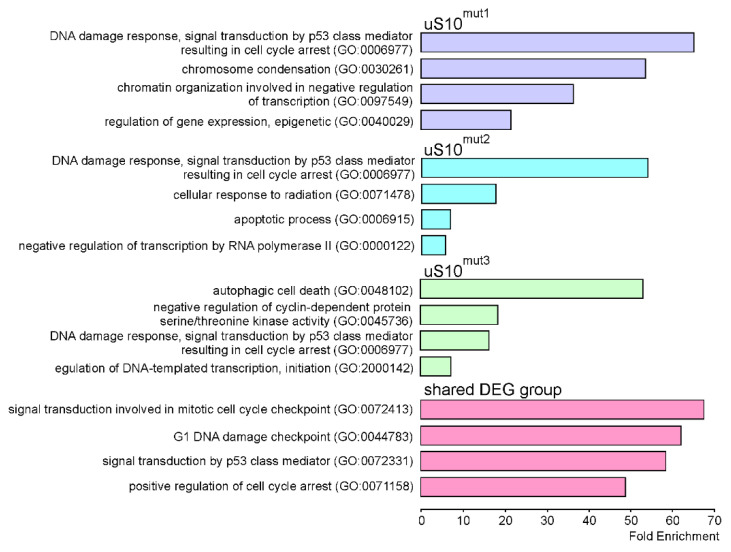
Gene Ontology (GO) enrichment analysis of biological processes for genes that are upregulated in HEK293T cells producing mutant forms of uS10^wt^. The top four highest processes in the hierarchy of GO biological ones with fold enrichment value of more than 5 are shown for the DEG sets defined for each of the mutants (uS10^mut1^, uS10^mut2^ or uS10^mut3^) and for the group of DEGs present in all of these sets (shared DEG group). GO terms and indexes are indicated on the left.

With DEGs identified for cells producing mutant forms of uS10^wt^, Gene Ontology (GO) enrichment analysis was performed to determine associated biological processes. It turned out that downregulated DEGs found for cells producing uS10^mut1^ or uS10^mut2^, which were very few (1 and 5, respectively), are engaged in diverse processes, including those such as signal transduction (GO:0007165, *NDRG1*), collagen fibril organization (GO:0030199, *CRTAP*), transcription initiation from RNA polymerase II promoter (GO:0006367, *NR2C2AP*) and mitochondrial respiratory chain complex I assembly (GO:0032981, *MT-ND6*). For uS10^mut3^-producing cells, where quite a lot of downregulated DEGs were found (Table S3), the analysis revealed only one process, namely collagen fibril organization (GO:0030199), for which the calculated fold enrichment value was greater than 5 (more or less) and with which 10 DEGs were associated, giving an enrichment value of 9.57. On the contrary, for upregulated DEGs, a very high enrichment was observed in a number of cellular processes (Figure 6), and one of them was the DNA damage response via signal transduction by p53 class mediator resulting in cell cycle arrest (GO:0006977), which was a common process for all three samples of cells producing the mutant protein. Among DEGs linked to this process, there are *GADD45A*, *PLK2*, *MDM2*, *CDKN1A*, and some others. Notably, the processes related to the above one were enriched with genes from the group of shared DEGs derived from overlapping DEG sets found for cells producing uS10^mut1^, uS10^mut2^ or uS10^mut3^. 

**Figure 7:**
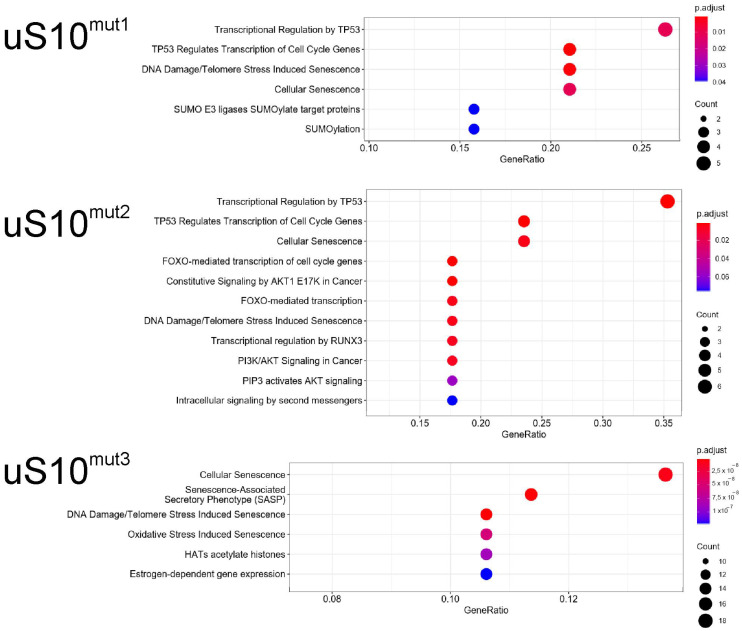
The Reactome pathway enrichment analysis of genes upregulated in cells produced mutant forms of uS10^wt^. Top significantly affected pathways are listed. The color of the dots corresponds to the value of p. adj, and their size is determined by the number of genes related to the respective pathway (map color keys, along with dot size ones, are shown on the right).

Reactome pathway analysis applied to the set of downregulated DEGs, found for cells producing uS10^mut3^, revealed that these DEGs were mainly enriched in pathways related to the extracellular matrix organization (e.g., *LAMA5*, *COL4A6* and *COL1A1*), the metabolism of carbohydrates (*ALDH1A1*, *ENO2*, *XYLT2* and other) and diseases of the metabolism (*AGRN*, *MOGS*, *NOTCH3* and other) (Table S5). In upregulated DEG sets, an enrichment was found for pathways mainly associated with transcriptional regulation by TP53 (*TP53INP1*, *CDKN1A*, *GADD45A*, *PLK2*, *MDM2* and other), cellular senescence (*MDM2*, *CDKN1B*, *H2BC11*, *JUN*, *H1-2* and other) and related processes for each of the three samples of cells producing the mutant protein (Figure 7, Table S5).

Thus, the gained results show that the production of mutated forms of uS10 by cells, regardless of whether the mutation is disruptive or missense, causes the activation of a limited set of genes. Many of these genes are involved in the regulatory processes through the p53 pathway and the induction of cellular senescence, although it should be borne in mind that the activity of p53 itself in HEK293T cells may be limited due to its interaction with the T antigen [49,50]. Along with them, genes implicated in cellular responses to events such as DNA damage, oxidative (or telomere) stress, the activation of cell cycle arrest, and autophagy, which are closely related to the above regulatory processes, are also activated. This means that mutations in the uS10 ribosomal protein gene resulting in the appearance of mutant forms of the protein, truncated ones (p.V50SfsX23 and p.L61EfsX11) or full-length one with a single substitution (p.V54L), affect the state of cells, forcing them to trigger processes that increase the risk of their malignant transformation.

## 3. Discussion

In this study, by applying RNA-seq to HEK293T cells ectopically expressing the uS10^wt^ ribosomal protein minigene or its forms with mutations associated with a predisposition to colorectal cancer, we examined the changes in the cellular transcriptome caused by the production of the respective aberrant proteins. We showed that site-directed mutations of the minigene, such as the insertion of an A residue into the Val50 codon and a double TT deletion in the Leu61 codon, leading to a frameshift, result in the accumulation in cells of the truncated forms of uS10, which are not incorporated in 40S ribosomal subunits. In contrast, the uS10 mutant form with the V54L missense mutation can be integrated into 40S subunits while retaining their functional activity. By analyzing RNA-seq data from DNA libraries, derived from total RNA samples isolated from HEK293T cells transfected with DNA constructs encoding the target proteins, we found differentially expressed genes between cells producing the above uS10^wt^ mutant forms and those producing uS10^wt^. Our findings revealed that the aberrant forms of uS10^wt^, regardless of the type of mutation and whether it interferes with the participation of the protein in the assembly of the 40S subunit, mainly cause the up regulation of limited sets of genes closely associated with the DNA damage response, the p53 pathway and carcinogenesis, which share a group of genes common for all three cell samples. Although some of the genes were also upregulated when cells produced exogenous uS10^wt^, the production of the uS10 mutants resulted in their more pronounced activation. Thus, it is not surprising that, as mentioned in the Introduction, mutations in the *RPS20* gene, which correspond to the disruptive mutations in the uS10 ribosomal protein described in the current study, can increase the risk of developing cancer. 

It would seem that the forms uS10^mut1^ and uS10^mut3^, which are unable to be incorporated into the 40S subunit, should not affect the pattern of protein synthesis in cells. The opposite effect observed with these forms could result from the appearance of misfolded, non-functional proteins in the cell, which are known to disrupt its proteome homeostasis, causing the unfolded protein response and senescence [51,52]. Furthermore, there is evidence of close links between the unfolded protein and DNA damage responses [53,54,55]. Hence, in cells producing inactive mutant forms of uS10^wt^, the activation of genes involved in the DNA damage response could be triggered through the responses to the emergence of these proteins and the need for their degradation. Notably, the GADD45a protein, whose gene was found to be upregulated in all cell samples producing mutant forms of uS10^wt^, has been recently discovered as a gene, mediating a potential link between the responses to the hypoxic DNA damage and unfolded proteins [55]. It is also noteworthy that the amounts of the truncated uS10^mut1^ and uS10^mut3^ proteins are significantly less than the amount of the full-length uS10^wt^ protein in cells transfected with the corresponding constructs, which indicates the active degradation of the former two. Meanwhile, it has been established that there is a direct relationship between aberrant protein folding and carcinogenesis [56,57], and that the increased degradation of misfolded proteins contributes to oncogenesis [58]. Therefore, the accumulation of an erroneous ribosomal protein in the cell, in particular a truncated form of uS10, may also contribute to carcinogenesis, especially because ribosomal proteins are synthesized in large quantities [11]. It should be noted that the frameshifts resulting from the p.L61EfsX11 and p.V50SfsX23 mutations in the *RPS20* gene should lead to the emergence of premature termination codons in the mRNA regions corresponding to the gene’s last exon and to the appearance in cells of the mRNAs that are unable to degrade by the NMD mechanism [59], thus enabling the synthesis of uS10 truncated forms. 

As mentioned in the Introduction, ribosome-free protein uS10 is a target for Mdm2 and thus participates in the regulation of the Mdm2-p53-MdmX network. Accordingly, it can be expected that the degradation of the truncated forms of uS10^wt^ occurs through their Mdm2-dependent ubiquitination. Thus, the increased activity of the *MDM2* gene observed with the uS10^mut1^ and uS10^mut3^ proteins is obviously required for their effective disposal from the cell. This, in turn, should lead to an imbalance in the Mdm2-p53 network, the role of which in the progression of many types of tumors, including CRC, is well known [60,61,62], and, consequently, to the activation of some genes of the p53 pathway and the induction of cellular senescence. Remarkably, the amount of DEGs in uS10^mut3^-producing cells is much greater than that in uS10^mut1^-producing ones, which could be due to the higher pI value for uS10^mut3^ (8.61) compared to the respective one for uS10^mut1^ (6.06) and, therefore, to the higher affinity of uS10^mut3^ for RNA. Since non-functional proteins should bind to RNA nonspecifically, one can assume that uS10^mut3^ is more toxic to cells than uS10^mut1^. Apparently, it is this property of uS10^mut3^ that is associated with changes in the expression of genes whose activities are not affected by the other two mutations of uS10^wt^, in particular with the activation of genes related to cellular senescence and the downregulation of genes involved in the extracellular matrix organization and diseases of metabolism processes.

**Figure 8:**
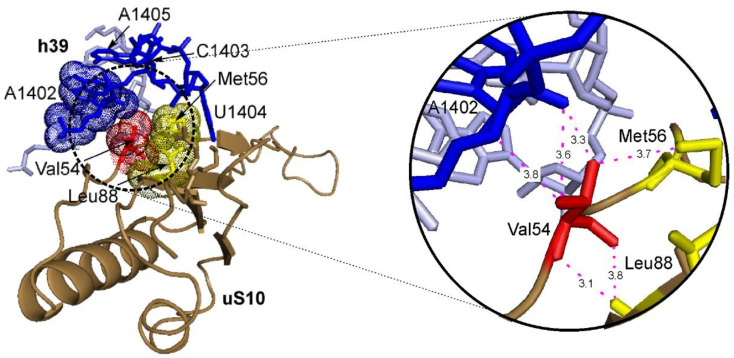
A fragment of the structure of the human ribosome ([64]; PDB: 6qzp) in the area of the contact of uS10 with the h39 helix of 18S rRNA. Nucleotide residues A1402–A1405 forming the pocket are shown in blue. On the left, amino acid residues Val54 (red), Met56 and Leu88 (both yellow) of uS10, which are grouped into a hydrophobic cluster, are indicated by arrows, and the van der Waals radii of their atoms are shown by dots. To display the tight contact between Val54 and A1402, their atoms are also depicted with van der Waals radii. On the right, an enlarged view of the outlined area of the left figure showing distances (in angstroms) between the Val54 atoms and key atoms of neighboring residues.

Interestingly, changes in the cellular transcriptome in response to the production of the uS10^mut2^ protein with the missense mutation, which could integrate into the 40S ribosomal subunit without disrupting its ability to participate in translation, were similar to those caused by the production of truncated proteins uS10^mut1^ and uS10^mut3^. However, the cellular response to the appearance of uS10^mut2^, in which, instead of the Val residue at position 54, there was the Leu residue with the same physicochemical properties as the Val one, could hardly be associated with the functional inactivity of the protein in any processes occurring outside the translation machinery. If we analyze the structure of the ribosome in the uS10 region, we can see that the Val54 residue together with the Met56 and Leu88 ones forms a hydrophobic cluster, which is located in a rather close environment of the 18S rRNA nucleotide residues A1402-A1405 in the h39 helix (Figure 8). Moreover, residues C1403 and U1404 are turned out of the helix and form a kind of pocket in which this cluster is located, while the ribose moiety of the A1402 residue contacts the Val54 side chain. Thus, the replacement of Val54 by a bulkier hydrophobic Leu residue probably leads to a distortion of the ribosome structure in this region, which, obviously, does not have a catastrophic effect on protein synthesis (as evidenced by the involvement of ribosomes containing uS10^mut2^ in translation). However, it can affect this process through the allosteric modulation of the ribosome structure in some other specific region. For example, uS10 comes into direct contact with the ribosomal protein uS3 (see Introduction), which, being near the mRNA entry site, can interact with the apurinic/apyrimidinic (AP) mRNA sites and, therefore, potentially participate in mRNA quality control during translation [7,63]. One can assume that this contact maintains the functionally active conformation of uS3 in the ribosome, which allows it to recognize an AP site in mRNA, and the distortion of the structure in the uS10 region prevents this contact, making uS3 unable to cross-link to the AP site. As a result of an increase in the number of errors during the decoding of mRNA codons containing the AP site, a significant number of non-functional proteins with amino acid substitutions should be synthesized, similarly to what occurs when mutations appear in damaged DNA, which implies that the cell responses to the impairment of mRNA quality control and the DNA damage are identical.

Notably, among the very limited number of upregulated DEGs in cells producing the uS10 mutants, there were genes directly associated with the progression of colorectal cancer. Of these, the *PPM1D* and *PIGN* genes have previously been identified as markers of colorectal cancer with a poor prognosis when highly expressed [65,66], whereas *PLK2* has been described as a gene that promotes tumor growth in colorectal cancer [67]. It is possible that the excessive activation of the above genes occurs also in patients with the mutations in the *RPS20* gene, which may contribute to their hereditary predisposition to CRC.

## 4. Materials and Methods

### 4.1. Plasmids Preparation, Cell Culturing and Target Protein Production Analysis

The minigene of the human ribosomal protein uS10 with the N-terminal 3×FLAG insertion was amplified using HEK293T cDNA and specific primers (Table S6). The plasmid pcDNA-uS10^wt^ was generated by inserting the above minigene into the pcDNA3.1 vector at the Bam HI site as described [68]. This plasmid was a template for preparing pcDNA-uS10^mut1^, pcDNA-uS10^mut2^ and pcDNA-uS10^mut3^ plasmids bearing targeted mutations in the CDS of the minigene (insertion, substitution and deletion, respectively) by the quick-change mutagenesis method, using specific primers (Table S6). To facilitate the screening of clones with target mutations, technical mutations (substitutions) that did not change the amino acid sequence of the protein were also introduced into the pcDNA-uS10^mut1^ and pcDNA-uS10^mut2^ plasmids. 

The HEK293T cells were cultured in 10 cm Petri dishes containing DMEM, 10% FBS, and 100 U/mL of penicillin-streptomycin in a CO_2_ incubator (5% CO_2_) at 37 °C. When reaching a density of 50–60%, the cells were transiently transfected with the above plasmids using Turbofect transfection reagent (Thermo Fisher Scientific, Waltham, MA, USA) according to the manufacturer’s protocol. After 48 h of incubation, the cells were washed with phosphate-buffered saline (PBS, Arlington, VA, USA), followed by washing them off the Petri dishes.

Cell lysis, a sucrose density gradient centrifugation of the lysate and an analysis of the 3×FLAG-tagged protein content in the gradient fractions and the cell lysate are described in [69]. Mouse monoclonal antibodies specific for FLAG-peptide (M2, #F1804 and M5, #F4042; Sigma, St. Louis, MO, USA), rabbit polyclonal antibodies specific for human uS10 (#PA5-112222; Thermo Fisher Scientific, Waltham, MA, USA) and mouse monoclonal antibodies specific for recombinant human GAPDH (#60004-1-Ig; Proteintech, Rosemont, IL, USA) were used for Western blotting.

### 4.2. DNA Libraries Preparation and High-Throughput Sequencing

Total RNA was isolated from the cells producing target proteins, and the quality of the obtained samples was checked on the Bioanalyzer 2100 using the RNA6000Pico kit (Agilent Technologies, Santa Clara, CA, USA). Total RNA was isolated from cells using TRIzol reagent with subsequent treatment by the On-Column DNase I Digestion Set (Sigma, St. Louis, MO, USA) and polyA enrichment by the NEBNext Poly(A) mRNA Magnetic Isolation Module (NEB, Ipswich, MA, USA).

DNA libraries were prepared from mRNA samples using the MGIEasy RNA Directional Library Prep Set (MGI Tech, Shenzhen, China) according to the manufacturer’s instructions and subjected to NGS on the MGISEQ-2000 platform, utilizing a sequencing set 2 × 100 PE (FCL PE100, MGI Tech). All relevant procedures were performed in SB RAS Genomics Core Facility (ICBFM SB RAS, Novosibirsk, Russia).

### 4.3. Raw NGS Data Processing

Raw reads in fastq formats were quality assessed using the FastQC (v.0.11.9) and MultiQC (v. 1.9) tools [70] and subjected to quality filtration (Trimmomatic 0.39 [71]) and adapter trimming (cutadapt 2.9 [72]) using adapter sequences provided by the manufacturer. The filtered reads were quality assessed and mapped to the hg38 reference human genome using the STAR RNA-seq aligner tool (2.7.3) [73] and the Ensembl annotation (release 102). The resulting BAM files were quality checked with the QualimapTool (v.2.2) [74] using default parameters. The generated quality metrics were collected in a metadata table. The analysis of gene coverage with the sequencing reads was performed in the IGV genome browser using the generated BAM files. The RNA-seq read data reported in this study were submitted to the GenBank under the study accession PRJNA738595. The validation of RNA-seq data by RT-qPCR was performed as described [75] using the appropriate gene-specific primers (Table S7).

### 4.4. Bioinformatics Analysis of Processed NGS Data

A table with raw read counts assigned to each gene (counts table) was prepared using the Rsubread package (v. 2.4.0) [76], utilizing the featureCounts function with the GTF file (Ensembl release 102) as an annotation in paired-end and reverse-stranded counting. The BioMart package (v. 2.46.0) [77] was applied to annotate genes with the HGNC symbol, entrez ID and description. The analysis of DEGs was performed using the DESeq2 (v. 1.30.0) [78] package with default parameters, and the apeglm algorithm was used to shrink the LFC values, as described in the package vignette. For the selection of DEGs, the *p* value adjusted (p.adj) cutoff was assigned to <0.1, the absolute shrunken LFC cutoff was assigned to >0.322, which corresponds to changes in expression levels of more than 25%, and the baseMean value was assigned to higher than 100, which excludes poorly covered genes, i.e., weakly expressed ones. Volcano plots were generated using the EnhancedVolcano package (v. 1.4.0).

The GO enrichment analysis of DEGs against the GO terms of the biological processes category was carried out using the online-based resource www.geneontology.org (accessed on 15 March 2021) [79] by selecting the Fisher’s Exact test type and False Discovery Rate (FDR) correction parameters. Pathway analysis was performed using the ReactomePA package (1.34.0) [80] with the p.adj cutoff parameter assigned to 0.1 and other parameters by default. Only the top pathways by the p.adj value were selected. The pI values of proteins were calculated using the web-based application https://web.expasy.org/compute_pi (accessed on 1 November 2021).

## 5. Conclusions

Studying the effects of ectopic synthesis of uS10 ribosomal protein forms with p.V50SfsX23, p.V54L or p.L61EfsX11 mutations, previously reported as associated with a predisposition to hereditary non-polyposis CRC, on the transcriptome profile of mammalian cells made it possible to determine the genes and processes that are activated upon the appearance of these forms in the cell. The presence of a number of genes related to the pathways involved in cellular responses to DNA damage and p53-regulation among the genes that are upregulated in all three studied samples of cells producing the mutant uS10 forms indicates the similarity in the mechanisms of responses to the synthesis of the aberrant proteins, and to stimuli promoting the emergence of cancer. Thus, our findings contribute to our understanding of the relationship between inherited mutations in the *RPS20* gene, corresponding to the above ones in uS10, and a predisposition to non-polyposis CRC. Further studies using various cell models, including colonic epithelial cells with the mutations introduced in the *RPS20* gene, will help answer the question of why these mutations promote the malignant transformation of colon cells.

## Data Availability

The RNA-seq read data reported in this study were submitted to the GenBank under the study accession PRJNA738595.

## Supplementary Materials

The following supporting information can be downloaded at: https://www.mdpi.com/article/10.3390/ijms23116174/s1.

## References

[1] Thomson E., Ferreira-Cerca S., Hurt E. (2013). Eukaryotic ribosome biogenesis at a glance. J. Cell Sci..

[2] Bassler J., Hurt E. (2019). Eukaryotic ribosome assembly. Annu. Rev. Biochem..

[3] Yamamoto H., Unbehaun A., Loerke J., Behrmann E., Collier M., Bürger J., Mielke T., Spahn C.M.T. (2014). Structure of the mammalian 80S initiation complex with initiation factor 5B on HCV-IRES RNA. Nat. Struct. Mol. Biol..

[4] Muhs M., Hilal T., Mielke T., Skabkin M.A., Sanbonmatsu K.Y., Pestova T.V., Spahn C.M. (2015). Cryo-EM of ribosomal 80S complexes with termination factors reveals the translocated cricket paralysis virus IRES. Mol. Cell.

[5] Simonetti A., Brito Querido J., Myasnikov A.G., Mancera-Martinez E., Renaud A., Kuhn L., Hashem Y. (2016). eIF3 peripheral subunits rearrangement after mRNA binding and start-codon recognition. Mol. Cell.

[6] Graifer D., Karpova G. (2015). Interaction of tRNA with eukaryotic ribosome. Int. J. Mol. Sci..

[7] Graifer D., Karpova G. (2020). Ribosomal protein uS3 in cell biology and human disease: Latest insights and prospects. Bioessays.

[8] Graifer D., Karpova G. (2021). Eukaryotic protein uS19: A component of the decoding site of ribosomes and a player in human diseases. Biochem. J..

[9] Venturi G., Montanaro L. (2020). How altered ribosome production can cause or contribute to human disease: The spectrum of ribosomopathies. Cells.

[10] Goudarzi K.M., Lindstrom M.S. (2016). Role of ribosomal protein mutations in tumor development. Int. J. Oncol..

[11] Warner J.R., McIntosh K.B. (2009). How common are extraribosomal functions of ribosomal proteins?. Mol. Cell.

[12] Bhavsar R.B., Makley L.N., Tsonis P.A. (2010). The other lives of ribosomal proteins. Hum. Genom..

[13] Graifer D., Malygin A., Zharkov D.O., Karpova G. (2014). Eukaryotic ribosomal protein S3: A constituent of translational machinery and an extraribosomal player in various cellular processes. Biochimie.

[14] Nieminen T.T., O’Donohue M.F., Wu Y., Lohi H., Scherer S.W., Paterson A.D., Ellonen P., Abdel-Rahman W.M., Valo S., Mecklin J.-P. (2014). Germline mutation of RPS20, encoding a ribosomal protein, causes predisposition to hereditary nonpolyposis colorectal carcinoma without DNA mismatch repair deficiency. Gastroenterology.

[15] Broderick P., Dobbins S.E., Chubb D., Kinnersley B., Dunlop M.G., Tomlinson I., Houlston R.S. (2017). Validation of recently proposed colorectal cancer susceptibility gene variants in an analysis of families and patients—A systematic review. Gastroenterology.

[16] Djursby M., Madsen M.B., Frederiksen J.H., Berchtold L.A., Therkildsen C., Willemoe G.L., Hasselby J.P., Wikman F., Okkels H., Skytte A.-B. (2020). New pathogenic germline variants in very early onset and familial colorectal cancer patients. Front. Genet..

[17] Anger A.M., Armache J.P., Berninghausen O., Habeck M., Subklewe M., Wilson D.N., Beckmann R. (2013). Structures of the human and Drosophila 80S ribosome. Nature.

[18] Khatter H., Myasnikov A.G., Natchiar S.K., Klaholz B.P. (2015). Structure of the human 80S ribosome. Nature.

[19] Cuchalova L., Kouba T., Herrmannova A., Danyi I., Chiu W.L., Valasek L. (2010). The RNA recognition motif of eukaryotic translation initiation factor 3g (eIF3g) is required for resumption of scanning of posttermination ribosomes for reinitiation on GCN4 and together with eIF3i stimulates linear scanning. Mol. Cell. Biol..

[20] Mitterer V., Shayan R., Ferreira-Cerca S., Murat G., Enne T., Rinaldi D., Weigl S., Omanic H., Gleizes P.-E., Kressler D. (2019). Conformational proofreading of distant 40S ribosomal subunit maturation events by a long-range communication mechanism. Nat. Commun..

[21] Higgins R., Gendron J.M., Rising L., Mak R., Webb K., Kaiser S.E., Zuzow N., Riviere P., Yang B., Fenech E. (2015). The unfolded protein response triggers site-specific regulatory ubiquitylation of 40S ribosomal proteins. Mol. Cell.

[22] Matsuo Y., Ikeuchi K., Saeki Y., Iwasaki S., Schmidt C., Udagawa T., Sato F., Tsuchiya H., Becker T., Tanaka K. (2017). Ubiquitination of stalled ribosome triggers ribosome-associated quality control. Nat. Commun..

[23] Sundaramoorthy E., Leonard M., Mak R., Liao J., Fulzele A., Bennett E.J. (2017). ZNF598 and RACK1 regulate mammalian ribosome-associated quality control function by mediating regulatory 40S ribosomal ubiquitylation. Mol. Cell.

[24] Ikeuchi K., Tesina P., Matsuo Y., Sugiyama T., Cheng J., Saeki Y., Tanaka K., Becker T., Beckmann R., Inada T. (2019). Collided ribosomes form a unique structural interface to induce Hel2-driven quality control pathways. EMBO J..

[25] Daftuar L., Zhu Y., Jacq X., Prives C. (2013). Ribosomal proteins RPL37, RPS15 and RPS20 regulate the Mdm2-p53-MdmX network. PLoS ONE.

[26] Krishnan R., Boddapati N., Mahalingam S. (2018). Interplay between human nucleolar GNL1 and RPS20 is critical to modulate cell proliferation. Sci. Rep..

[27] McGowan K.A., Li J.Z., Park C.Y., Beaudry V., Tabor H.K., Sabnis A.J., Zhang W., Fuchs H., de Angelis M.H., Myers R.M. (2008). Ribosomal mutations cause p53-mediated dark skin and pleiotropic effects. Nat. Genet..

[28] Bhar S., Zhou F., Reineke L.C., Morris D.K., Khincha P.P., Giri N., Mirabello L., Bergstrom K., Lemon L.D., Williams C.L. (2020). Expansion of germline RPS20 mutation phenotype to include Diamond-Blackfan anemia. Hum. Mutat..

[29] Kampen K.R., Sulima S.O., Vereecke S., De Keersmaecker K. (2020). Hallmarks of ribosomopathies. Nucleic Acids Res..

[30] Salvador J.M., Brown-Clay J.D., Fornace A.J. (2013). Gadd45 in stress signaling, cell cycle control, and apoptosis. Adv. Exp. Med. Biol..

[31] Schafer A. (2013). Gadd45 proteins: Key players of repair-mediated DNA demethylation. Adv. Exp. Med. Biol..

[32] Tamura R.E., de Vasconcellos J.F., Sarkar D., Libermann T.A., Fisher P.B., Zerbini L.F. (2012). GADD45 proteins: Central players in tumorigenesis. Curr. Mol. Med..

[33] Emelyanov A., Bulavin D.V. (2017). Wip1 phosphatase in breast cancer. Oncogene.

[34] Deng W., Li J., Dorrah K., Jimenez-Tapia D., Arriaga B., Hao Q., Cao W., Gao Z., Vadgama J., Wu Y. (2020). The role of PPM1D in cancer and advances in studies of its inhibitors. Biomed. Pharmacother..

[35] Li K., Liu Y., Xu S., Wang J. (2020). PPM1D functions as oncogene and is associated with poor prognosis in psophageal squamous cell carcinoma. Pathol. Oncol. Res..

[36] Yin Z., Yi J., Nie T., Yang Z., Ding N., Du S.X., Liu S., Peng T. (2019). Silencing of PPMD1 inhibits proliferation of human colon cancer cells via induction of apoptosis and cell cycle arrest. J. Buon..

[37] Raab C.A., Raab M., Becker S., Strebhardt K. (2021). Non-mitotic functions of polo-like kinases in cancer cells. Biochim. Biophys. Acta Rev. Cancer.

[38] Han J., Zhang L., Zhang J., Jiang Q., Tong D., Wang X., Gao X., Zhao L., Huang C. (2018). CREBRF promotes the proliferation of human gastric cancer cells via the AKT signaling pathway. Cell. Mol. Biol..

[39] Xue H., Zhang J., Guo X., Wang J., Li J., Gao X., Guo X., Li T., Xu S., Zhang P. (2016). CREBRF is a potent tumor suppressor of glioblastoma by blocking hypoxia-induced autophagy via the CREB3/ATG5 pathway. Int. J. Oncol..

[40] Fahraeus R., Olivares-Illana V. (2014). MDM2’s social network. Oncogene.

[41] Wade M., Li Y.C., Wahl G.M. (2013). MDM2, MDMX and p53 in oncogenesis and cancer therapy. Nat. Rev. Cancer.

[42] Oliner J.D., Saiki A.Y., Caenepeel S. (2016). The Role of MDM2 amplification and overexpression in tumorigenesis. Cold Spring Harb. Perspect. Med..

[43] Kitamura N., Nakamura Y., Miyamoto Y., Miyamoto T., Kabu K., Yoshida M., Futamura M., Ichinose S., Arakawa H. (2011). Mieap, a p53-inducible protein, controls mitochondrial quality by repairing or eliminating unhealthy mitochondria. PLoS ONE.

[44] Dan X., Babbar M., Moore A., Wechter N., Tian J., Mohanty J.G., Croteau D.L., Bohr V.A. (2021). DNA damage invokes mitophagy through a pathway involving Spata18. Nucleic Acids Res..

[45] Tomasini R., Samir A.A., Pebusque M.J., Calvo E.L., Totaro S., Dagorn J.C., Dusetti N.J., Iovanna J.L. (2002). P53-dependent expression of the stress-induced protein (SIP). Eur. J. Cell Biol..

[46] Ito Y., Motoo Y., Yoshida H., Iovanna J.L., Nakamura Y., Kuma K., Miyauchi A. (2006). High level of tumour protein p53-induced nuclear protein 1 (TP53INP1) expression in anaplastic carcinoma of the thyroid. Pathology.

[47] Taïeb D., Giusiano S., Sebag F., Marcy M., De Micco C., Palazzo F.F., Dusetti N.J., Iovanna J.L., Henry J.F., Garcia S. (2010). Tumor protein p53-induced nuclear protein (TP53INP1) expression in medullary thyroid carcinoma: A molecular guide to the optimal extent of surgery?. World J. Surg..

[48] Giusiano S., Garcia S., Andrieu C., Dusetti N.J., Bastide C., Gleave M., Taranger-Charpin C., Iovanna J.L., Rocchi P. (2012). TP53INP1 overexpression in prostate cancer correlates with poor prognostic factors and is predictive of biological cancer relapse. Prostate.

[49] Lin Y.C., Boone M., Meuris L., Lemmens I., Roy N.V., Soete A., Reumers J., Moisse M., Plaisance S., Drmanac R.T. (2014). Genome dynamics of the human embryonic kidney 293 lineage in response to cell biology manipulations. Nat. Commun..

[50] Sheppard H.M., Corneillie S.I., Espiritu C., Gatti A., Liu X. (1999). New insights into the mechanism of inhibition of p53 by Simian virus 40 large T antigen. Mol. Cell. Biol..

[51] Van Drie J.H. (2011). Protein folding, protein homeostasis, and cancer. Chin. J. Cancer.

[52] Pluquet O., Pourtier A., Abbadie C. (2011). The unfolded protein response and cellular senescence. A review in the theme: Cellular mechanisms of endoplasmic reticulum stress signaling in health and disease. Am. J. Physiol. Cell Physiol..

[53] Gorgoulis V.G., Pefani D.E., Pateras I.S., Trougakos I.P. (2018). Integrating the DNA damage and protein stress responses during cancer development and treatment. J. Pathol..

[54] Dufey E., Pedro J.M.B.-S., Eggers C., González-Quiroz M., Urra H., Sagredo A.I., Sepulveda D., Pihán P., Carreras-Sureda A., Hazari Y. (2020). Genotoxic stress triggers the activation of IRE1alpha-dependent RNA decay to modulate the DNA damage response. Nat. Commun..

[55] Bolland H., Ma T.S., Ramlee S., Ramadan K., Hammond E.M. (2021). Links between the unfolded protein response and the DNA damage response in hypoxia: A systematic review. Biochem. Soc. Trans..

[56] Scott M.D., Frydman J. (2003). Aberrant protein folding as the molecular basis of cancer. Methods Mol. Biol..

[57] Kato H., Nishitoh H. (2015). Stress responses from the endoplasmic reticulum in cancer. Front. Oncol..

[58] Chen L., Brewer M.D., Guo L., Wang R., Jiang P., Yang X. (2017). Enhanced degradation of misfolded proteins promotes tumorigenesis. Cell Rep..

[59] Lindeboom R.G., Supek F., Lehner B. (2016). The rules and impact of nonsense-mediated mRNA decay in human cancers. Nat. Genet..

[60] Meng X., Franklin D.A., Dong J., Zhang Y. (2014). MDM2-p53 pathway in hepatocellular carcinoma. Cancer Res..

[61] Liebl M.C., Hofmann T.G. (2021). The role of p53 signaling in colorectal cancer. Cancers.

[62] Karni-Schmidt O., Lokshin M., Prives C. (2016). The roles of MDM2 and MDMX in cancer. Annu. Rev. Pathol..

[63] Ochkasova A.S., Meschaninova M.I., Venyaminova A.G., Ivanov A.V., Graifer D.M., Karpova G.G. (2019). The human ribosome can interact with the abasic site in mRNA via a specific peptide of the uS3 protein located near the mRNA entry channel. Biochimie.

[64] Natchiar S.K., Myasnikov A.G., Hazemann I., Klaholz B.P. (2018). Visualizing the role of 2’-OH rRNA methylations in the human ribosome structure. Biomolecules.

[65] Peng T.-S., He Y.-H., Nie T., Hu X.-D., Lu H.-Y., Yi J., Shuai Y.-F., Luo M. (2014). PPM1D is a prognostic marker and therapeutic target in colorectal cancer. Exp. Ther. Med..

[66] Uhlen M., Zhang C., Lee S., Sjöstedt E., Fagerberg L., Bidkhori G., Benfeitas R., Arif M., Liu Z., Edfors F. (2017). A pathology atlas of the human cancer transcriptome. Science.

[67] Ou B., Zhao J., Guan S., Wangpu X., Zhu C., Zong Y., Ma J., Sun J., Zheng M., Feng H. (2016). Plk2 promotes tumor growth and inhibits apoptosis by targeting Fbxw7/Cyclin E in colorectal cancer. Cancer Lett..

[68] Yanshina D.D., Gopanenko A.V., Karpova G.G., Malygin A.A. (2020). Replacement of hydroxylated His39 in ribosomal protein uL15 with Ala or Thr impairs the translational activity of human ribosomes. Mol. Biol..

[69] Babaylova E.S., Gopanenko A.V., Bulygin K.N., Tupikin A.E., Kabilov M.R., Malygin A.A., Karpova G.G. (2020). mRNA regions where 80S ribosomes pause during translation elongation in vivo interact with protein uS19, a component of the decoding site. Nucleic Acids Res..

[70] Ewels P., Magnusson M., Lundin S., Kaller M. (2016). MultiQC: Summarize analysis results for multiple tools and samples in a single report. Bioinformatics.

[71] Bolger A.M., Lohse M., Usadel B. (2014). Trimmomatic: A flexible trimmer for Illumina sequence data. Bioinformatics.

[72] Martin M. (2011). Cutadapt removes adapter sequences from high-throughput sequencing reads. EMBnet. J..

[73] Dobin A., Davis C.A., Schlesinger F., Drenkow J., Zaleski C., Jha S., Batut P., Chaisson M., Gingeras T.R. (2013). STAR: Ultrafast universal RNA-seq aligner. Bioinformatics.

[74] Okonechnikov K., Conesa A., Garcia-Alcalde F. (2016). Qualimap 2: Advanced multi-sample quality control for high-throughput sequencing data. Bioinformatics.

[75] Gopanenko A.V., Kolobova A.V., Meschaninova M.I., Venyaminova A.G., Tupikin A.E., Kabilov M.R., Malygin A.A., Karpova G.G. (2021). Knockdown of the mRNA encoding the ribosomal protein eL38 in mammalian cells causes a substantial reorganization of genomic transcription. Biochimie.

[76] Liao Y., Smyth G.K., Shi W. (2019). The R package Rsubread is easier, faster, cheaper and better for alignment and quantification of RNA sequencing reads. Nucleic Acids Res..

[77] Durinck S., Spellman P.T., Birney E., Huber W. (2009). Mapping identifiers for the integration of genomic datasets with the R/Bioconductor package biomaRt. Nat. Protoc..

[78] Love M.I., Huber W., Anders S. (2014). Moderated estimation of fold change and dispersion for RNA-seq data with DESeq2. Genome Biol..

[79] Gene Ontology Consortium (2021). The Gene Ontology resource: Enriching a GOld mine. Nucleic Acids Res..

[80] Yu G., He Q.Y. (2016). ReactomePA: An R/Bioconductor package for reactome pathway analysis and visualization. Mol. Biosyst..

