# Supplementary material for: Changes in the Transcriptome Caused by Mutations in the Ribosomal Protein uS10 Associated with a Predisposition to Colorectal Cancer
Source: Int J Mol Sci. 2022 May 31;23(11):6174. doi: 10.3390/ijms23116174 (PMC9181716; doi:10.3390/ijms23116174)
Supplement: Supplementary file 1 [file ijms-23-06174-s001.zip › ijms-1724764-supplementary/Supplementary Material.pdf]

Supplementary Material

Supplementary Figures

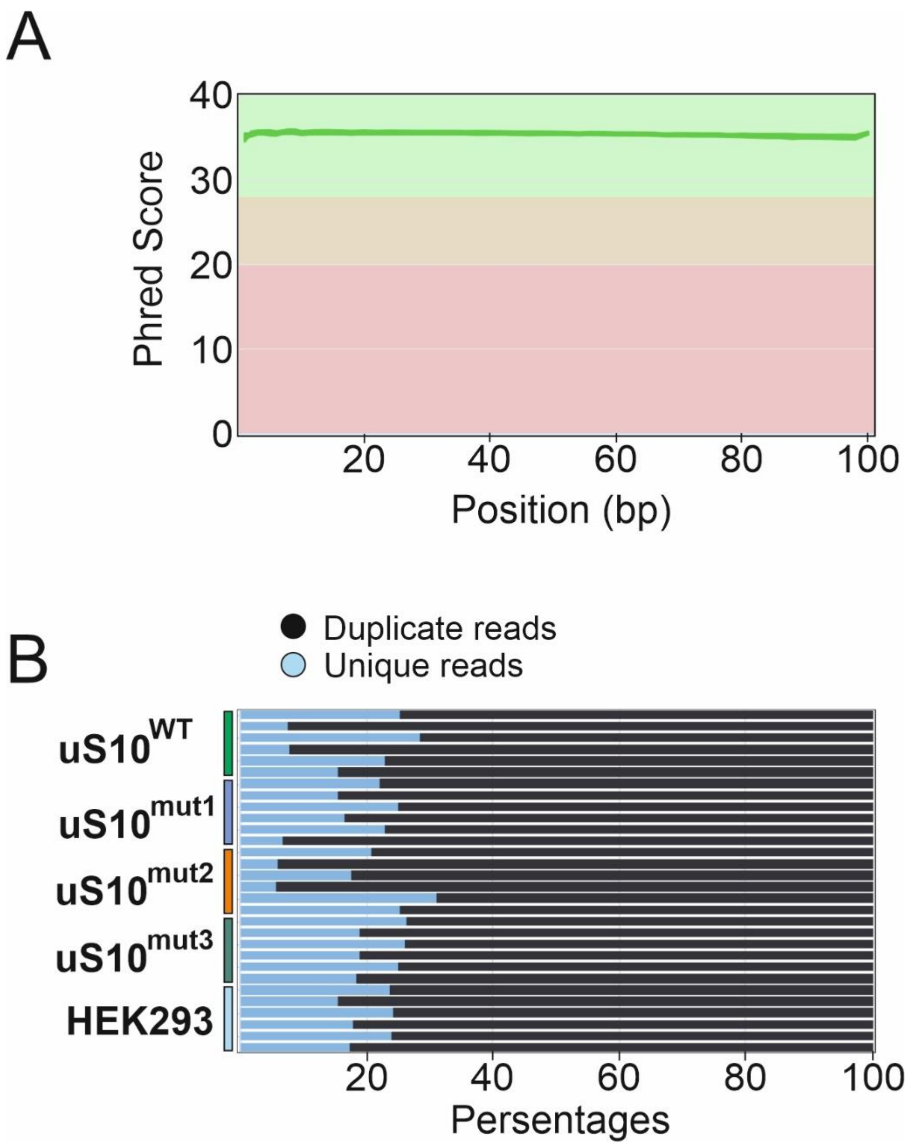

Supplementary Figure S1. A quality assessment of the filtered fastq data. **(A)** The MultiQC-derived sequence quality histograms for each sample generated based on individual FastQC reports of fastq files after quality filtering and adapters trimming. **(B)** The MultiQC-derived duplicate reads estimation for each sample.

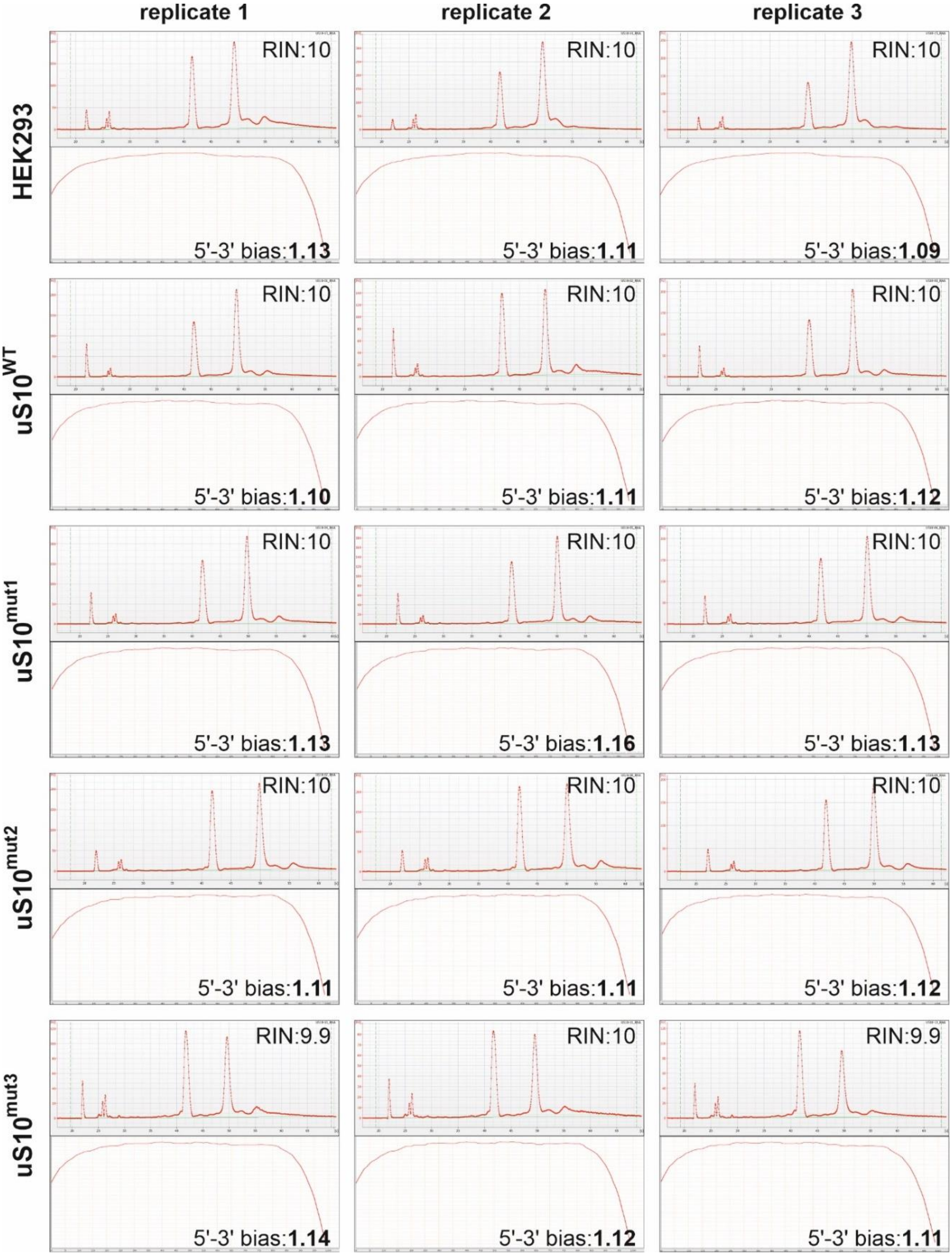

Supplementary Figure S2. A quality assessment of RNA samples. The upper panels for each sample represent the Bioanalyzer 2100 (Agilent RNA 6000 Pico) RNA quality report. Each panel is supplied with an RIN (RNA integrity number) value. The bottom panels represent the coverage profiles across transcripts (according to Qualimap 2 reports) for each sample. Each panel is supplied with 5’-3’ bias value.

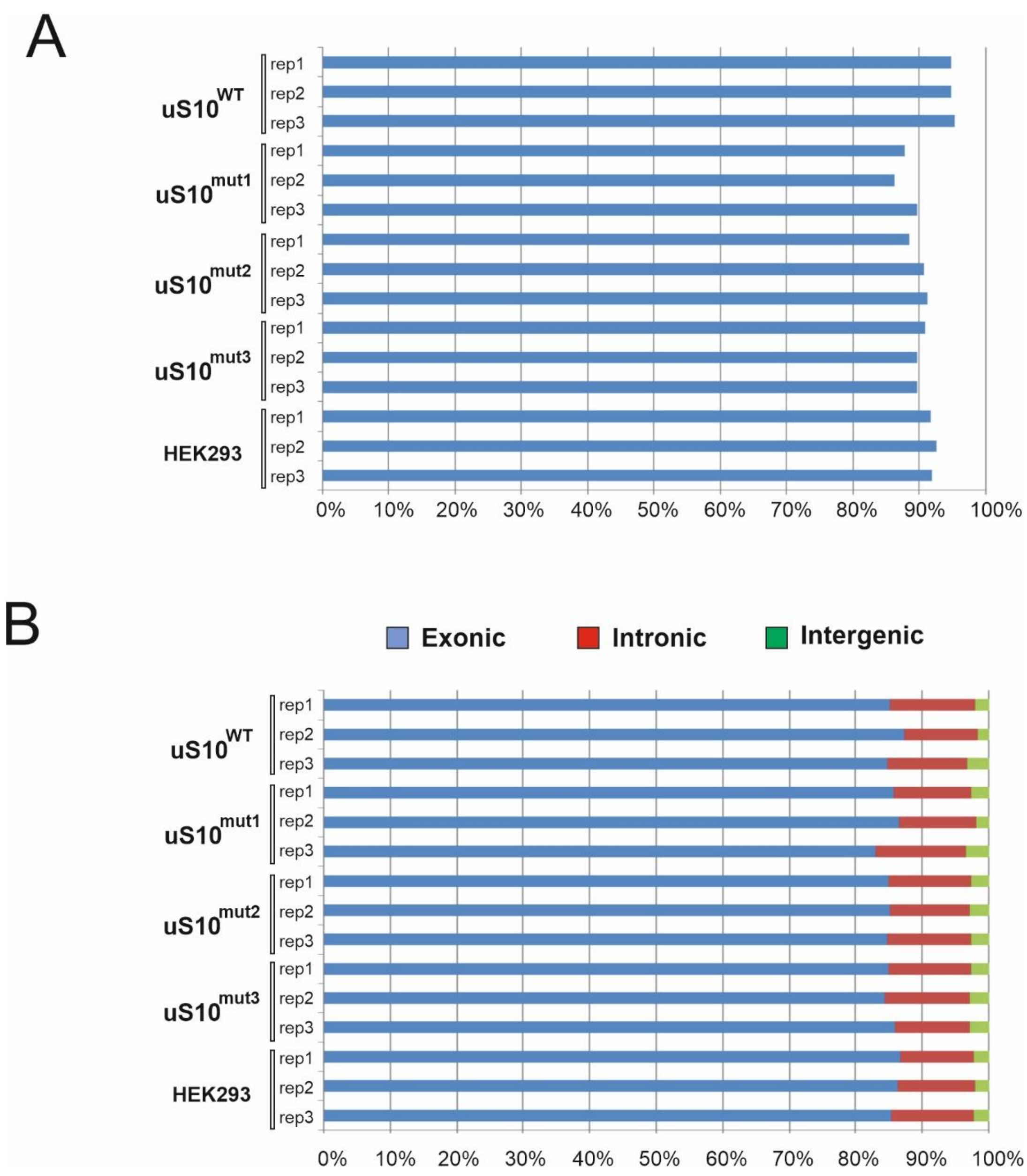

Supplementary Figure S3. Statistics of sequencing reads mapping to genome. **A.** The percent of uniquely mapped reads (according to the STAR final report) for each library. **B.** The distribution of aligned reads across genomic features (according to Qualimap 2 reports).

Supplementary Tables

Supplementary Table S1. Metadata with characteristics of each library. The Excel table.

Supplementary Table S2. Pairwise sorting of genes with changes in expression levels in cells producing uS10^{mut1}, uS10^{mut2} or uS10^{mut3}, compared to uS10^{wt}-producing ones predicted by the DESeq2 package. The Excel table.

Supplementary Table S3. Statistically relevant DEGs determined for HEK293T cells producing mutant uS10^{wt} forms. The Excel table.

Supplementary Table S4. Statistically relevant DEGs determined for HEK293T cells producing uS10^{wt}. The Excel table.

Supplementary Table S5. Results of the Reactome analysis of pathways associated with down-regulated and up-regulated DEGs. The Excel table.

Supplementary Table S6. The list of primers used for uS10^{wt} DNA cloning and mutagenesis.

**primer**	**sequence (5'-3')**
F-uS10-BamHI	caacggatccatggactacaaagaccatgacggtgattataaagatcatgacatcgactac
F-uS10-FLAG	aaagatcatgacatcgactacaaagacgatgacgacaaggcttttaaggataccg
R-BamHI-uS10	ctgcggattcttaagcatcttgcaatggtgacttccacctc
F-V50S-uS10	cgcaaaagaaaagaatctcaaaagtgagcgcaccagttcgaatgcctac
R-V50S-uS10	gtaggcattcgaactggtgcgctcacttttgagattcttttcttttgcg
F-V54L-uS10	gaaaagaatctcaaagtgaaaggcccacttcgaatgcctaccaagactttg
R-V54L-uS10	caaagtcttggtaggcattcgaagtgggcctttcactttgagattcttttc
F-L61E-uS10	gttcgaatgcctaccaagactgagaatcactacaagaaaaac
R-L61E-uS10	gtttttcttgtagtgattctcagtcttggtaggcattcgaac

Supplementary Table S7. The set of oligonucleotides used for RT-qPCR.

**gene**	**forward primer (5'-3')**	**reverse primer (5'-3')**
PLK2	tttcaacaatggtgctcacatg	agccactgaaggaggtagag
PPM1D	ctccagaagtggacaatcag	gactacaccttggacattctc
GADD45A	ggagagcagaagaccgaaag	ggcacaacaccacgttatc
TP53INP1	tcttgagtgcttggctgatac	gtggggtgataaaccagctc
MDM2	atgccattgaaccttgtgtg	ggttgtctacatactgggca
GAPDH	gtgaaccatgagaagtatgacaac	catgagtccttccacgatacc
